# Time-dependent treatment effects of metronomic chemotherapy in unfit AML patients: a secondary analysis of a randomised controlled trial

**DOI:** 10.1186/s13104-020-05423-5

**Published:** 2021-01-06

**Authors:** Phichayut Phinyo, Jayanton Patumanond, Saranya Pongudom

**Affiliations:** 1 Department of Family Medicine and Center for Clinical Epidemiology and Clinical Statistics, Faculty of Medicine, Chiang Mai University, Chiang Mai, Thailand; 2 Faculty of Medicine, Center for Clinical Epidemiology and Clinical Statistics, Chiang Mai University, Chiang Mai, Thailand; 3 Department of Medicine, Udon Thani Medical Education Center, Udon Thani Hospital, Udon Thani, Thailand

**Keywords:** Leukemias, Maintenance chemotherapy, Survival, Proportional hazard model

## Abstract

**Objectives:**

To examine the presence of the time-dependent effect of metronomic chemotherapy for the treatment of older patients with acute myeloid leukemia (AML) who were unfit for standard chemotherapy and to reanalyze the data using an appropriate statistical approach in the presence of non-proportional hazards, the restricted mean survival time (RMST).

**Results:**

This was a secondary analysis of a multi-center, open-label, randomized controlled trial, which was conducted in seven tertiary care hospitals across Thailand. A total of 81 unfit AML patients were randomized into two treatment groups, metronomic chemotherapy and palliative treatment. The hazard ratio of metronomic chemotherapy over palliative treatment was time-dependent. At three landmark time points of 90, 180, 365 days, the restricted mean survival time differences were 13.3 (95% CI 1.9–24.7) days, 28.9 (95% CI 3.3–54.4) days, and 40.4 (95% CI − 1.3 to 82.0) days, respectively. With non-proportional hazards modeling and RMST analysis, we were able to conclude that metronomic chemotherapy is a potentially effective alternative treatment for elderly AML patients who were medically unfit for intensive chemotherapy. In the future clinical trials, non-proportional hazards should be carefully inspected and properly handled with appropriate statistical methods.

*Trial registration* Randomized clinical trial TCTR20150918001; registration date: 15/09/2015. Retrospectively registered

## Introduction

Proper therapeutic choices for elderly patients with acute myeloid leukemia (AML) remains controversial [1]. The median survival of the untreated AML patients was reported at about two to three months [2]. In resource-limited countries, including Thailand, palliative treatment is generally the mainstay of treatment in this patient domain. During the past decades, metronomic chemotherapy, or the administration of low-dose chemotherapy without a prolonged drug-free period, has appeared as a suitable treatment strategy to control advanced malignancy, as it is more tolerable and can be practically applied with low cost [3].

The efficacy and safety of metronomic chemotherapy in AML patients who were unfit for standard chemotherapy were addressed for the first time in our prior work [4]. It was revealed that the overall survival was higher in patients who were allocated to metronomic chemotherapy compared to those allocated to palliative treatment. However, the decrease in treatment effect was observed as the survival curves finally merged. This could suggest the violation of proportional hazards (PH) assumption. In this situation, reporting a single hazard ratio (HR) is also misleading, as there was evidence that the treatment effect is time-dependent [5].

Several methods have been proposed for the analysis of clinical trials with departure from PH assumption [6, 7]. Restricted mean survival time (RMST) has recently been paid more attention in recent literature due to its statistical robustness against non-PH and its clinical interpretability [5, 8, 9]. Even though the method seems attractive and has been advocated by many experts, it is still underutilized. The main objective of this secondary analysis is to follow an alternative approach of trial analysis for estimation of time-varying treatment effects in a situation where the PH assumption is unlikely to hold, by the use of non-PH modeling and RMST.

## Main text

### Methods

The trial was a multi-center randomized controlled trial, which was conducted in seven tertiary care hospitals across Thailand. Patients aged ≥ 55 years with a histologically-confirmed diagnosis of AML based on WHO definitions who either refused or were considered unfit for intensive chemotherapy were enrolled into the study, starting from December 2014 to December 2017. Included patients were randomly allocated to one of the two treatments: metronomic chemotherapy or palliative treatment.

Patients in the metronomic chemotherapy arm were given a low and sustained dose of oral chemotherapy regimen as follows: 50 mg per m^2^ of etoposide for 5 days, together with 60 mg per m^2^ of 6-mercaptopruine and 40 mg per m^2^ of prednisolone for two weeks. The regimen was administered every three weeks for four cycles. Patients in the palliative treatment arm were given standard care with oral hydroxyurea. The primary outcome was overall survival (OS), which was defined as the time since randomization until death from any cause. The remaining information on the trial design, patient eligibility (inclusion and exclusion criteria), schedule of appointed follow-up, and outcome measurements were described in our previous report [4].

#### Statistical analysis

All statistical analyses were performed with Stata 16 (StataCorp, Texas, USA). Frequency and percentages were used to describe categorical data. Mean and standard deviation or median and interquartile range were used to describe continuous data as appropriate. In this re-analysis, we estimated the treatment effect with Kaplan–Meier survival curves and the log-rank test. Then, the HR of OS was estimated from Cox’s PH regression. The Grambsch–Therneau test was used to test for evidence of non-PH of treatment effect.

We employed the flexible parametric regression, *stpm2* command in Stata [10], for modeling differences in the RMST and allowing for time-dependent treatment effect. In this study, we assigned 3 d.f. for the baseline hazard distribution and 1 d.f. for modeling treatment-time interaction as suggested by Royston [11]. To illustrate the time-dependent effect, the HR was estimated and plotted as a function of time with the flexible parametric model. The analysis of RMST and the RMST difference between the treatment groups was performed with *strmst* command in Stata [12]. As the calculation of RMST requires pre-specified time point (t*), we chose three-time points as follow: 90 days, 180 days, and 365 days. The latter two choices were based on previous report [4], whereas 90 days was based on the median survival time of unfit AML patients [2, 13]. Finally, we presented the changes in RMST and the difference in RMST as a function of time to visualize how treatment effect changes over time.

### Results

A total of 81 patients, who were diagnosed as unfit AML, were included in the intention-to-treat analysis, 40 in metronomic chemotherapy arm and 41 in the palliative treatment arm. About 60% of the patients were female with a median age of 66 years. There were no significant difference in baseline clinical characteristics between groups.

**Fig. 1:**
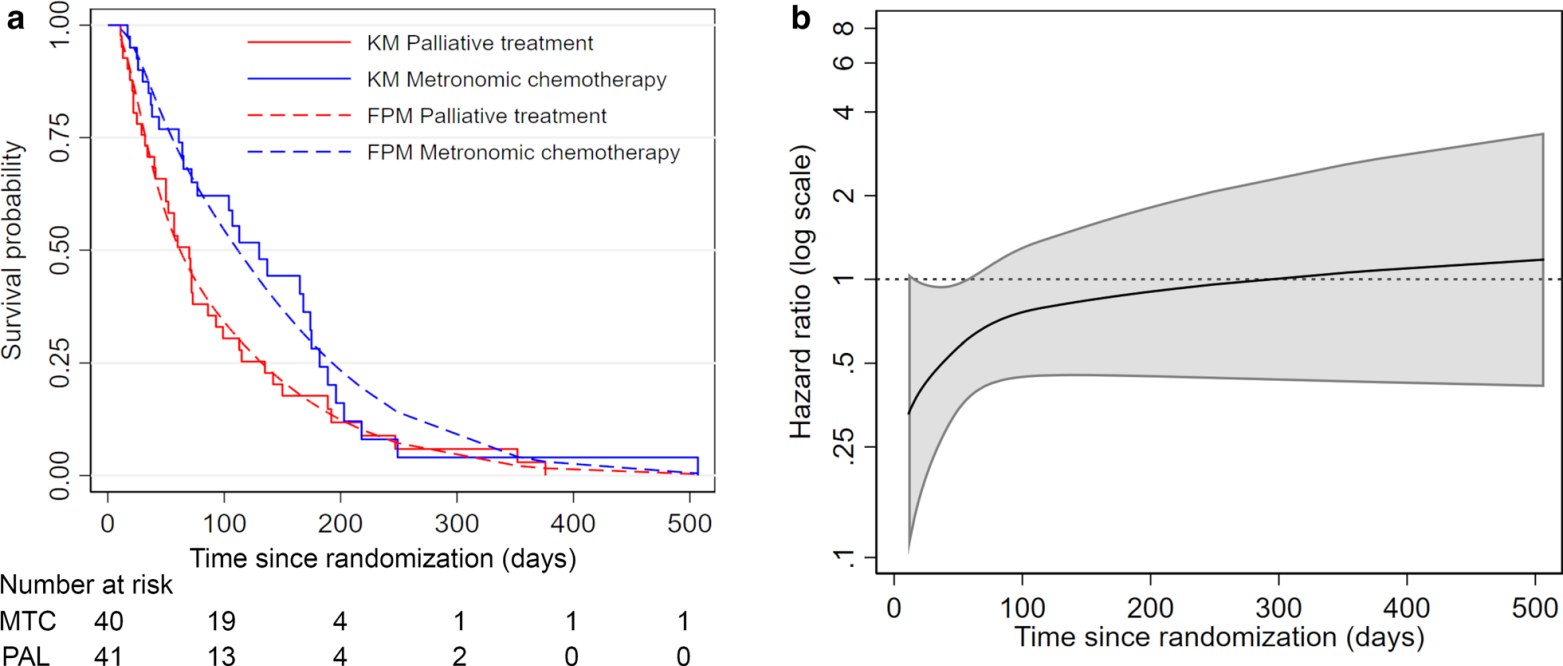
Evidence of non-proportional hazards and time-dependent hazard ratio. **a** Kaplan–Meier survival estimates (KM) and flexible parametric model survival estimates (FPM) showed decreasing treatment effect. **b** Time-dependent hazard ratio and 95% confidence intervals estimated from flexible parametric model. *MTC* metronomic chemotherapy, *PLA* palliative treatment

At the end of the trial, a total number of 69 (85.2%) events occurred. The median survival time was 130 (95% CI 64–115) and 70 (95% CI 41–93) days in the metronomic chemotherapy and palliative treatment arm, respectively. For the treatment effect in terms of OS, the Kaplan–Meier curves by treatment arm were shown in Fig. 1a. The survival curves showed an early divergence of patients’ survival until about 180 days. However, both survival curves merged in the later part of the follow-up. The p value from the log-rank test was 0.073. The estimated HR for OS from the Cox’s PH model was 0.65 (95% CI 0.40–1.05, p = 0.077).

**Fig. 2:**
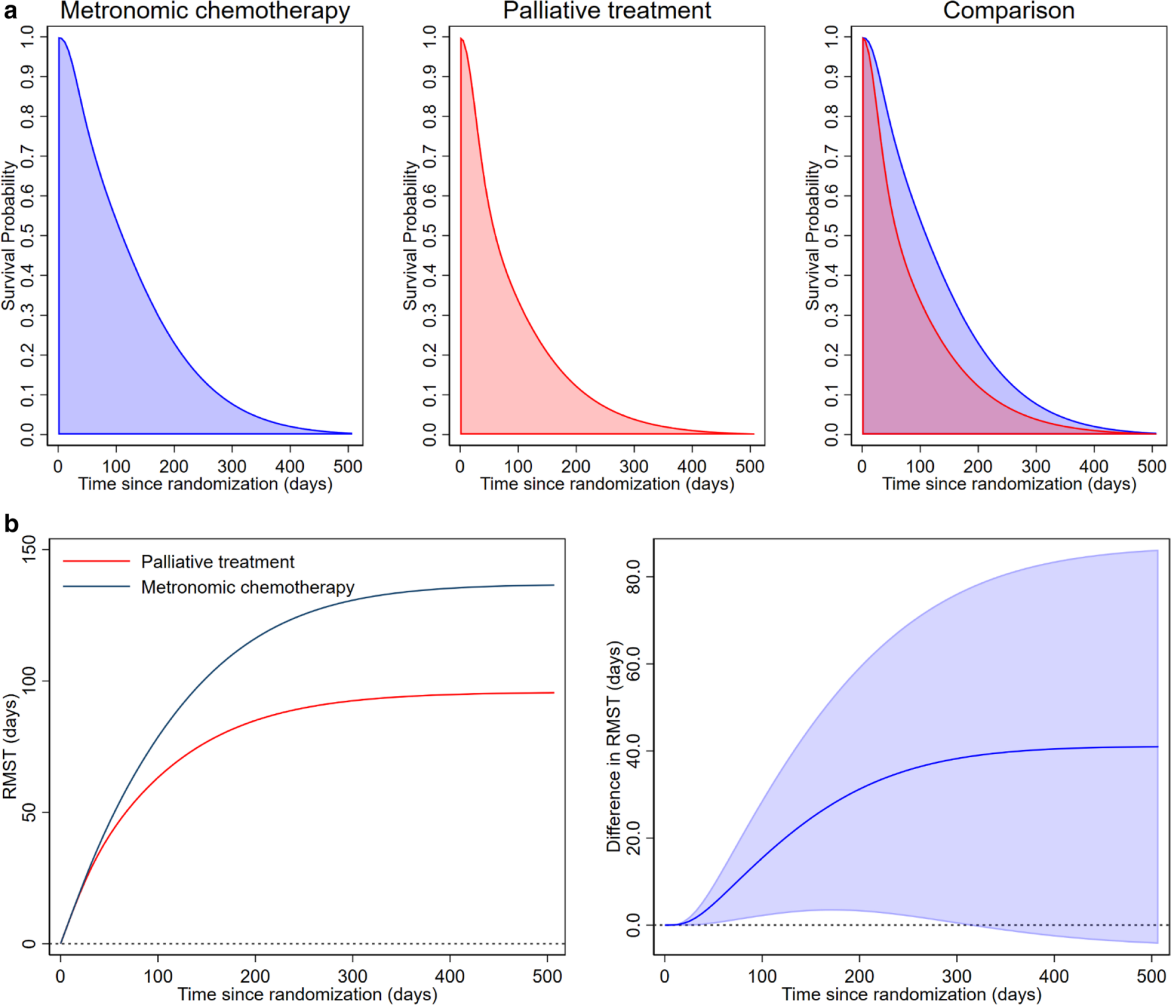
Visualization of restricted mean survival time analysis. **a** Restricted mean survival time over the follow-up period as the area under the survival curve. [metronomic chemotherapy (blue) vs. palliative treatment (red)], **b** Restricted mean survival time changes (left) and the difference in restricted mean survival time changes (right) as a function of time

According to the Grambsch–Therneau test, no statistical evidence of non-PH of treatment effect was identified (p = 0.356). With flexible parametric modeling, the HR and its confidence interval were estimated as a function of time to examine the presence of time-varying effects. The result was shown in Fig. 1b. It was observed that the HR crosses 1 (i.e., line of no treatment effect) at about 300 days after randomization or around the end of the first year. This decrement in treatment effect over time would suggest non-PH. The results of the RMST analyses were shown in Table 1 (Fig. 2a). The RMST and the difference in RMST as a function of time were illustrated in Fig. 2b.

**Table 1:** Restricted mean survival time (RMST) differences between treatment groups at three pre-specified landmarks (t*)

Landmark t* (days)	Metronomic chemotherapy (n = 40)		Palliative treatment (n = 41)		Treatment effect	p value
	Number at risk	RMST (95% CI)	Number at risk	RMST (95% CI)	RMST Difference (95% CI)	
90	20	73.0 (65.3–80.7)	14	59.7 (50.9–68.5)	+ 13.3 (1.9 to 24.7)	0.022
180	7	111.1 (92.3–129.8)	6	82.2 (64.4–100.0)	+ 28.9 (3.3 to 54.4)	0.027
365	1	134.2 (101.5–166.9)	1	93.9 (68.0–119.7)	+ 40.4 (− 1.3 to 82.0)	0.058

*CI* confidence interval, *RMST* restricted mean survival time, t* pre-specified time points for estimating the restricted mean survival time

### Discussion

The results of this secondary analysis confirmed the primary efficacy endpoint of our previous study by showing the extension of 90-day and 180-day mean survival time by about two and four weeks, respectively. These seemingly-modest survival improvements are considered clinically-significant based on a low median survival of the patients at around three months. This study also supported our prior hypothesis that the treatment effect of metronomic chemotherapy might be time-dependent or non-PH.

The strengths of our study lay within the methods we used for analysis. First, this secondary analysis employed the appropriate statistical approach analysis of time-to-event outcomes with non-PH. From our previous study, no statistical evidence of non-PH was identified. However, the absence of evidence was not the evidence of PH, as the tests of PH assumption usually were underpowered, especially in small studies [7, 14]. Second, the RMST was chosen over other non-PH methods as it was more suitable for treatment with early or diminished effect, as in our case [7]. It was evident from the previous re-analysis of a trial examining the progression-free survival of pediatric patients with solid malignant tumors that the analysis of RMST was able to identify the statistical significance in the presence of non-PH [15]. Third, the reporting of RMSTs and their differences also give a clinically-meaningful interpretation of trial results compared to the HR [16, 17]. Although some authors recently opposed the interpretability of RMST [6], we still did not find that one single HR would have any clinical meaning or direct interpretation, especially in the presence of non-PH.

The results of our study should raise the awareness of inappropriate analysis of time-to-event clinical endpoints. PH assumption should always be examined before the conventional analysis using the log-rank test and Cox’s PH model. Relying solely on statistical tests for the detection of non-PH might not be adequate. We suggest that Kaplan–Meier curves must be carefully inspected. If the distance between curves was not proportionate or the curve crossed, diverged, or merged, non-PH should be suspected. In the case where non-PH is obvious, it is unreasonable to rely on the PH model. Alternative statistical methods in the presence of non-PH should be incorporated in the study protocol and stated in statistical analytic plans [7].

### Conclusions

With appropriate statistical methods for the analysis of time-to-event with non-PH, we were able to conclude that metronomic chemotherapy is a potentially effective alternative treatment for elderly AML patients who were medically unfit for intensive chemotherapy. The RMST-based approach should be applied more often in the clinical trial community to improve the statistical robustness and clinical interpretability, especially when non-PH is evident.

### Limitations

There were also some limitations to be addressed. Firstly, this was the post-hoc analysis of the previously reported clinical trial, where the analysis and study size estimation were based on the PH assumption. Therefore, the analysis of RMST might not be adequately powered. Secondly, the major limitation of the RMST method is that it requires the pre-specified time point (t*) for the integration of the area under the survival curves. The selection of t* heavily influences the statistical significance of RMST and could result in biased or exaggerated RMST estimates [6, 18]. However, in this analysis, the selection of t* was based on our previous study. In addition, these time points were generally used as points of follow-up in many oncologic trials. Thirdly, the analysis and reporting of RMST without proper consideration of the Kaplan–Meier curves might be misleading. As in the previously mentioned example of one trial of pediatric solid malignant tumors [19], the survival curves did not show any meaningful separation along the course of follow-up and only showed a significant difference in the end. This was in contrast to our study, where the survival curves showed significant divergence during the early period of follow-up. The merging of survival curves after six months might be explained by the variation in patients’ susceptibility to mortality [14, 20]. As susceptible patients in the palliative treatment arm were rapidly depleted early in the study period, the remaining survivors beyond six months carried relatively lower mortality risk compared to the survivors in the metronomic arm.

## Data Availability

The datasets used and/or analysed during the current study are available from the corresponding author on reasonable request.

## Abbreviations

AML: Acute myeloid leukemia
PH: Proportional hazards
HR: Hazard ratio
RMST: Restricted mean survival time
WHO: World health organization
OS: Overall survival
